# The Rise of SARS-CoV-2 (COVID-19) Omicron Subvariant Pathogenicity

**DOI:** 10.7759/cureus.40148

**Published:** 2023-06-08

**Authors:** David C DeGrasse, Shaun D Black

**Affiliations:** 1 Chemistry and Biochemistry, University of Texas at Tyler, Tyler, USA

**Keywords:** public health or epidemiology studies, mrna vaccines, chlorpheniramine maleate, antiviral therapy, case fatality rate (cfr), xbb.1.5, omicron variants, sars-cov-2 virus, covid-19

## Abstract

During the COVID-19 pandemic, variants of the *Betacoronavirus* SARS-CoV-2, the etiologic agent of COVID-19 disease, progressively decreased in pathogenicity up to the Omicron strain. However, the case fatality rate has increased from Omicron through each major Omicron subvariant (BA.2/BA.4, BA.5, XBB.1.5) in the United States of America. World data also mirror this trend. We show that the rise of Omicron pathogenicity is exponential, and we have modeled the case fatality rate of the next major subvariant as 0.0413, 2.5 times that of the Alpha strain and 60% of the original Wuhan strain which caused the greatest morbidity and mortality during the pandemic. Small-molecule therapeutics have been developed, and some of these, such as chlorpheniramine maleate, may be useful in the event of an Omicron subvariant of higher risk.

## Introduction

The coronavirus pandemic of 2019-2023 has caused over 6,880,000 deaths in over 676,600,000 confirmed cases worldwide by early 2023 [1]. The etiologic agent of COVID-19 disease is the *Betacoronavirus* SARS-CoV-2, first identified in late 2019 in Wuhan, China [2-4]. Variants emerged that were more infectious and supplanted earlier forms, namely Alpha, Beta, Delta, and Omicron; pathogenicity and case fatality rate (CFR) decreased progressively and reached a minimum with the Omicron variant [5]. At this point early in 2022, the pandemic was said to be over, and that the endemic phase of COVID-19 had begun [6].

Subvariants of the Omicron strain arose, each more infectious than the previous ones [1,3,5,6]. Major subvariants included Omicron BA.2/BA.4 (mid-2022), Omicron BA.5 (late 2022), and Omicron XBB.1.5 (early 2023) [5]. The latter is a recombinant of Omicron BA.2.10.1 and BA.2.75 [7]. It was expected that the CFR of these subvariants should have decreased as did all previous forms, but this has not proven to be the case [1,5]. Here, we report an exponential increase in the pathogenicity of these later Omicron subvariants, and we model the CFR of the next major form of SARS-CoV-2 Omicron.

## Materials and methods

Confirmed COVID-19 case rates per 100,000 population and COVID-19 deaths per 100,000 population in the United States of America were obtained from the Centers for Disease Control for all major forms of SARS-CoV-2 [8]. Peak values of each of these were determined, and respective maxima were found to be out of phase, as expected; phase corrections were applied by moving the time of respective peak cases to that of corresponding peak deaths. CFR was then calculated as $\frac{peak \ confirmed \ Deaths \ per \ 100,000}{peak \ confirmed \ Cases \ per \ 100,000}$ for the original Wuhan strain and for Alpha, Beta, Delta, and Omicron variants and for each Omicron subvariant. These data were plotted and modeled mathematically in MS Excel. We examined data both phenomenologically and temporally; the former considered only existence of each viral form, whereas the latter examined the date of peak deaths for each during the pandemic. Both of these cases were modeled linearly, exponentially, logarithmically, and polynomially.

CFR values for the Omicron strain and successor subvariants were evident as an upward trend, and we examined linear, exponential, logarithmic, and polynomial functions to model this behavior. For linear fitting, slopes and intercepts were varied; for exponential modeling, coefficients, exponents, and intercepts were varied; for logarithmic fitting, coefficients, *ln* term, and intercepts were varied; and for polynomial modeling, coefficients, and degree were varied. The Pearson R-Factor was calculated to show goodness-of-fit quantitatively in each case. Exponential modeling calculations are available in the supplemental information of the appendix.

## Results

Primary epidemiologic data and case fatality rate calculation results are shown in Table 1. 

**Table 1:** Primary Data and Calculated Case Fatality Rates (CFR) of SARS-CoV-2 and Variants in the United States of America. Case and mortality data were obtained from the US Centers for Disease Control (CDC).

Virus, variant, or subvariant	Confirmed cases per 100,000	Confirmed deaths per 100,000	CFR
Wuhan strain (SARS-CoV-2)	65.5	4.71	0.0719
Alpha variant	138.0	2.27	0.0164
Beta variant	518.0	7.12	0.0137
Delta variant	355.0	4.39	0.0124
Omicron variant	1658.0	5.27	0.0032
Omicron BA.2/BA.4 subvariant	230.0	0.76	0.0033
Omicron BA.5 subvariant	279.0	1.14	0.0041
Omicron XBB.1.5 subvariant	145.0	1.24	0.0086

**Figure 1:**
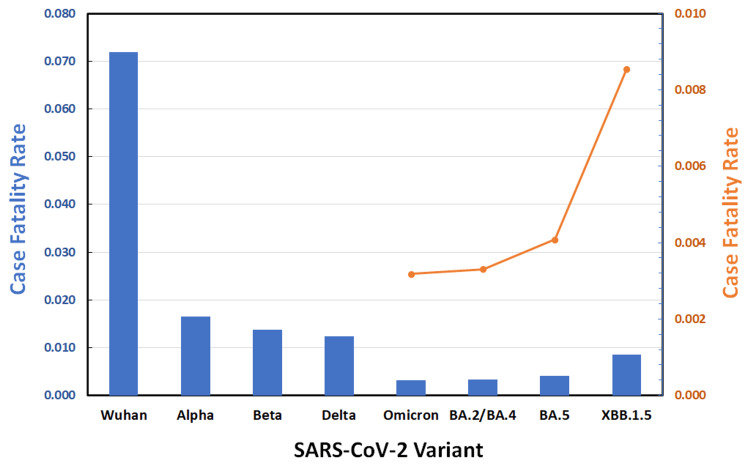
Case Fatality Rate (CFR) of SARS-CoV-2 Variants. CFR of blue bars refer to the left-hand y-axis, whereas the orange CFR inset refers to the right-hand y-axis with an expanded scale for Omicron and its subvariants. Variants are indicated on the x-axis; subvariants of the Omicron strain are indicated to the right of this variant.

The CFR for all major forms of SARS-CoV-2 is shown in Figure 1 as blue bars. The original Wuhan strain caused the greatest CFR (0.0719), but the Alpha variant was responsible for the highest mortality (7.12 deaths/100,000 population) in the United States. CFR and pathogenicity decreased from variant to variant until Omicron, when it reached a minimum (CFR = 0.0032). 

Unexpectedly, the case fatality rate began to rise from the parent Omicron strain through subvariants BA.2/BA.4, BA.5, and XBB.1.5. The CFR of Omicron XBB.1.5 (0.0086) is close to that of the Delta strain (0.0124). The expanded scale of the orange inset in Figure 1 shows that the increase from Omicron subvariant to subvariant is not linear but, instead, appeared to approximate exponential growth.

**Figure 2:**
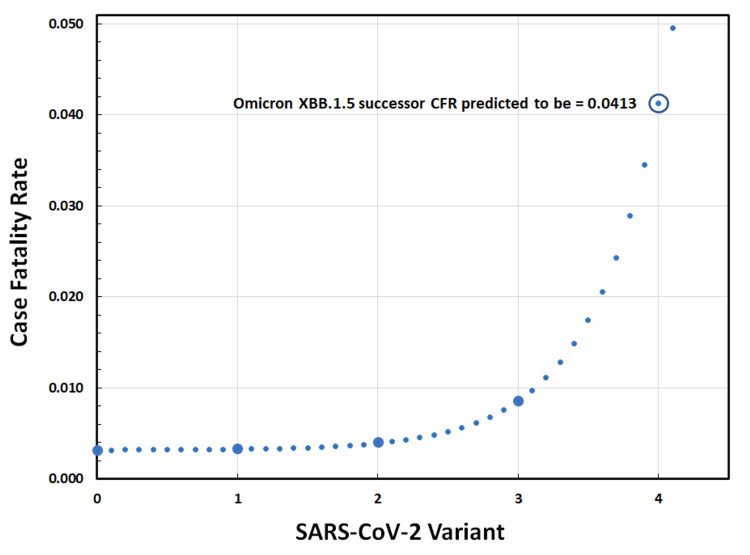
Modeling the Case Fatality Rate (CFR) of SARS-CoV-2 Omicron and Subvariants Phenomenologically. 0 = Omicron strain, 1 = BA.2/BA.4 subvariants, 2 = BA.5 subvariant, and 3 = XBB.1.5 subvariant. Large circles represent the actual CFR of Omicron and subsequent variants. An optimal exponential fit to these data is shown with small circles; the equation of this optimal curve is CFR = [(1.50 x 10^-5^) *e*^1.96(variant)^] + 3.15 x 10^-3^. Extrapolation of this function to the next putative Omicron-subvariant yielded a CFR = 0.0413, as shown inset in the figure.

We examined linear, exponential, logarithmic, and polynomial functions to fit the above results, but R^2^ values were poor in the case of linear, logarithmic, and polynomial functions; in contrast, only a positive exponential function fit the data well. The nature of the above Omicron trend is examined as an exponential in Figure 2. This exponential model fits the observed CFR of Omicron and its subvariants with R^2^= 0.9992. Because the goodness-of-fit was excellent, we extrapolated the curve to the putative next major Omicron variant and found a predicted CFR of 0.0413. This value is 2.5 times greater than that of the Alpha variant and is nearly 60% of the original Wuhan strain CFR.

**Figure 3:**
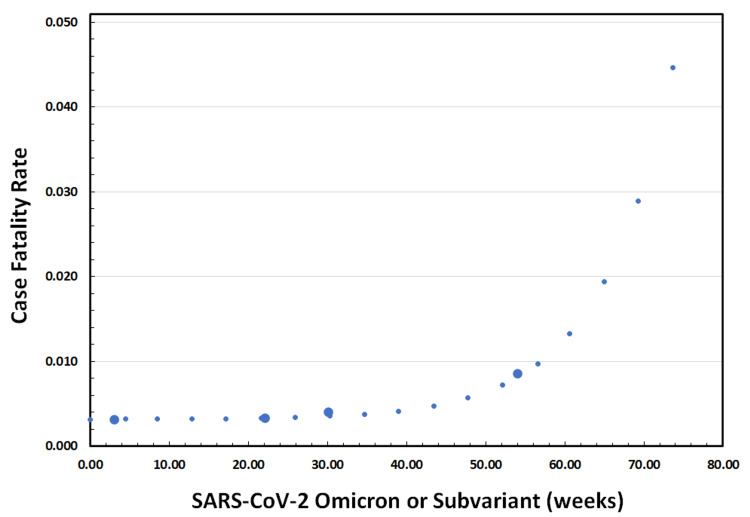
Modeling the Case Fatality Rate (CFR) of SARS-CoV-2 Omicron and Subvariants over Time. Omicron strain (peak 1/22/2021) at 3 weeks; BA.2/BA.4 subvariants (peak 6/4/2022) at 22 weeks; BA.5 subvariant (peak 7/30/2022) at 30 weeks; and XBB.1.5 subvariant (peak 1/14/2023) at 54 weeks. Large circles represent the actual CFR of Omicron and subsequent subvariants. An optimal exponential fit to these data is shown with small circles; the equation of this optimal curve is CFR = [(1.50 x 10^-5^) *e*^0.1075(time)^] + 3.15 x 10^-3^.

We also modeled the CFR of COVID-19 with respect to time, as shown in Figure 3. This temporal behavior was similar to the phenomenological model above, but the overall fit of the best-fit exponential was slightly lower at R^2^ = 0.9930. That is, the time-dependent findings of Figure 3 parallel those presented in Figure 2; the equations are also similar but differ in the exponent term.

## Discussion

The recent trend of SARS-CoV-2 Omicron subvariants contradicts previous behavior in which pathogenicity decreased from the Wuhan strain through the Omicron variant. That is, the CFR increased from Omicron to the XBB.1.5 subvariant. We show that this increase fits a positive exponential curve with excellent goodness-of-fit (R^2^ = 0.9992); modeled temporally, the fit is almost as good (R^2^ = 0.9930). Though results fit well in both cases, the phenomenological model is superior to the temporal model; this makes sense because viral evolution is mutation-driven, not time-dependent. We also note that many natural processes, including those in infectious disease, are exponential in nature [9].

The CFR of the XBB.1.5 subvariant has risen to pathogenicity near that of the Delta strain; world data also show a significant “death spike” due to Omicron XBB.1.5 during the week of January 15, 2023 (102% of Delta) [1]. Furthermore, when we extrapolated the phenomenological model to the next putative Omicron subvariant, we found a value of 0.0413, much higher than the Alpha variant, that caused most deaths during the pandemic, and nearly 60% as great as the CFR of the original Wuhan strain, which was the deadliest. Because this CFR value is on par with the Wuhan strain and Alpha variant, the worst previous forms, caution is advised. We considered extrapolation of the temporal model to CFR = 0.0413 to predict the date of this dangerous Omicron successor, but judged this unreliable as the extrapolation of an extrapolation.

Extrapolation is fraught with unavoidable error, and this is a limitation of our study; however, we note that swings in the functions during exponential curve fitting placed the predicted CFR of the Omicron successor near 0.04, regardless. The excellent goodness-of-fit of both models (R^2^ $\geq$ 0.9930) suggests that the error associated with extrapolation has been minimized. Also, extrapolation error increases with the distance from the last data point; our extrapolation is sensible, being only 25% from the XBB.1.5 value. Thus, though extrapolation can lead to difficulties, the quality of our results suggests that the predicted CFR (0.0413) of the successor Omicron subvariant is of reasonable accuracy.

Vaccines could offer protection against this potentially dangerous successor Omicron strain. However, recent data show that COVID-19 vaccines have diminished efficacy, even those updated with the Omicron spike-protein sequence [10,11]. In addition, COVID-19 mRNA vaccines have caused significant morbidity, long COVID, and the highest mortality in the history of vaccines [5]; these findings cast doubt that vaccines can protect us from a potentially deadly threat in the future.

Fortunately, much work has been performed on small-molecule therapeutics during the COVID-19 pandemic, and we can draw upon this knowledge at this time for help should a more pathogenic Omicron successor arise. Effective prescription drugs include hydroxychloroquine with zinc [5,12,13] and ivermectin [5,14]; over-the-counter drugs include chlorpheniramine maleate [5,15-16], a repurposed antihistamine; and nutraceuticals include glycyrrhizin [17] and quercetin with zinc [13,18]. Of these, the most accessible and cost-effective alternative is chlorpheniramine maleate.

## Conclusions

Present results show that SARS-CoV-2 Omicron subvariants have increased in pathogenicity to XBB.1.5 which has a CFR near that of the Delta strain. Recent increases in CFR are exponential, and we fit these data to a positive exponential function with high confidence (R^2^ = 0.9992). Extrapolation to the next putative Omicron subvariant suggested that the CFR should be in the vicinity of 0.04, which would make it more dangerous than the Alpha variant and 60% as lethal as the original Wuhan strain. Though we hope that this does not come to pass, caution is yet warranted at this time. Small-molecule therapeutics like chlorpheniramine maleate may offer adequate protection against increasingly pathogenic subvariants of the SARS-CoV-2 coronavirus.

## Appendices

AnExcel* *spreadsheet, "Rise of COVID-19 Omicron CFR modeling US data.xlsx" contains all of the calculations and exponential modeling carried out and presented in this report. This file is available from the corresponding author (Dr. Shaun D. Black ) upon request.
