# Correction: The Rise of SARS-CoV-2 (COVID-19) Omicron Subvariant Pathogenicity

**DOI:** 10.7759/cureus.c122

**Published:** 2023-06-14

**Authors:** David C DeGrasse, Shaun D Black

**Affiliations:** 1 Chemistry and Biochemistry, University of Texas at Tyler, Tyler, USA

This article has been corrected at the request of the authors to correct the URLs for references 13 and 14 in the initial published version. They have been updated from https://c19hcq.org/ to https://c19early.org/z and https://c19ivm.org/, respectively. The authors deeply regret that these errors were not identified and addressed prior to publication.

